# Supplementary material for: Whole‐Genome Resequencing Reveals Exceptions to Panmixia in the Exploited Top Fish Predator Antarctic Toothfish (Dissostichus mawsoni)
Source: Ecol Evol. 2026 Oct 19;16(10):e74262. doi: 10.1002/ece3.74262 (PMC13680862; doi:10.1002/ece3.74262)
Supplement: Supplementary file 1 [file ECE3-16-e74262-s001.pdf]

Supplementary Tables

Table S1. Group sizes (n) across datasets.

**3RAD dataset**	subarea 48.1	subarea 48.2	subarea 48.4
Subarea totals	40	41	28
		Dataset total	**108**
**5× WGR dataset**	area 48	area 58	area 88
pre-2000	1	1	6
post-2000	6	3	7
Area totals	**7**	**4**	**13**
		Dataset total	**24**
**2× WGR dataset**	area 48	area 58	area 88
pre-2000	1	1	10
post-2000	10	7	8
Area totals	**11**	**8**	**18**
		Dataset total	**37**
**noDS WGR dataset**	area 48	area 58	area 88
pre-2000	1	1	11
post-2000	12	7	9
Area totals	**13**	**8**	**20**
		Dataset total	**41**

Table S2. 3RAD sample metadata including management subarea, latitude, longitude, total length (cm), final coverage, and whether the sample was found to be an outlier in PCAs. All samples were caught in the Weddell Sea (area 48) in 2019, and derived from tissue.

Sample	Management subarea	Latitude	Longitude	Length (cm)	Coverage	PCA outlier?
190	48.1	-62.16	-54.07	87.0	32.61	no
191	48.1	-62.16	-54.07	147.0	36.05	no
192	48.1	-62.16	-54.07	71.0	36.01	no
193	48.1	-62.16	-54.07	157.0	33.62	no
194	48.1	-62.16	-54.07	152.0	35.05	no
195	48.1	-62.16	-54.07	82.0	31.69	no
196	48.1	-62.16	-54.07	137.0	28.04	no
197	48.1	-62.16	-54.07	112.0	35.20	no
198	48.1	-62.16	-54.07	140.0	34.89	no
199	48.1	-62.16	-54.07	82.0	30.37	no
200	48.1	-62.37	-53.50	105.0	33.05	no
201	48.1	-62.37	-53.50	115.0	45.61	no
203	48.1	-62.37	-53.50	78.0	28.14	no
204	48.1	-62.37	-53.50	98.0	46.68	**yes**
206	48.1	-62.37	-53.50	127.0	29.42	no
207	48.1	-62.37	-53.50	155.0	39.68	no
208	48.1	-62.37	-53.50	142.0	26.50	no
209	48.1	-62.37	-53.50	119.0	35.69	no
210	48.1	-62.37	-53.50	158.0	31.59	no
211	48.1	-62.37	-53.50	156.0	33.09	no
212	48.1	-62.37	-53.50	158.0	36.07	**yes**
213	48.1	-62.37	-53.50	101.0	31.36	no
214	48.1	-62.37	-53.50	115.0	36.61	no
215	48.1	-62.37	-53.50	114.0	25.12	no
216	48.1	-62.37	-53.50	161.0	31.25	no
217	48.1	-62.37	-53.50	136.0	30.51	no
218	48.1	-62.37	-53.50	105.0	31.06	no
219	48.1	-62.37	-53.50	105.0	29.02	no
223	48.1	-62.37	-53.50	122.0	31.51	no

Table S2 (continued)

Sample	Management subarea	Latitude	Longitude	Length (cm)	Coverage	PCA outlier?
224	48.1	-62.37	-53.50	156.0	32.93	no
225	48.1	-62.37	-53.50	60.0	30.55	no
226	48.1	-62.37	-53.50	136.0	39.39	no
228	48.1	-62.37	-53.50	165.0	41.44	no
229	48.1	-62.37	-53.50	143.0	33.20	no
230	48.1	-62.37	-53.50	158.0	36.92	no
231	48.1	-62.37	-53.50	158.0	33.08	no
232	48.1	-62.37	-53.50	147.0	34.50	no
233	48.1	-62.37	-53.50	125.0	38.57	no
234	48.1	-62.37	-53.50	138.0	35.11	no
235	48.1	-62.37	-53.50	66.0	38.39	no
238	48.2	-59.82	-34.92	156.0	33.13	no
240	48.2	-59.82	-34.92	168.0	29.93	no
241	48.2	-59.82	-34.92	166.0	28.51	no
242	48.2	-59.82	-34.92	160.0	31.70	no
243	48.2	-59.82	-34.92	153.0	30.56	no
244	48.2	-59.82	-34.92	131.0	29.51	no
247	48.2	-61.55	-38.77	120.0	27.82	no
248	48.2	-61.55	-38.77	152.0	30.04	no
249	48.2	-61.55	-38.77	148.0	30.09	no
250	48.2	-61.55	-38.77	161.0	29.91	no
251	48.2	-61.55	-38.77	129.0	32.42	no
252	48.2	-61.55	-38.77	154.0	30.34	no
253	48.2	-61.55	-38.77	151.0	32.16	no
254	48.2	-61.55	-38.77	140.0	25.68	no
258	48.2	-61.40	-34.74	149.0	28.41	no
259	48.2	-61.40	-34.74	152.0	30.17	no
260	48.2	-61.40	-34.74	156.0	28.50	no
261	48.2	-61.40	-34.74	152.0	35.00	no
262	48.2	-61.40	-34.74	149.0	34.49	no
263	48.2	-61.40	-34.74	163.0	32.49	no
264	48.2	-61.40	-34.74	158.0	28.27	no

Table S2 (continued)

Sample	Management subarea	Latitude	Longitude	Length (cm)	Coverage	PCA outlier?
265	48.2	-61.40	-34.74	163.0	35.71	no
266	48.2	-61.40	-34.74	158.0	27.74	no
267	48.2	-61.40	-34.74	159.0	35.05	no
268	48.2	-61.40	-34.74	146.0	29.03	no
269	48.2	-61.40	-34.74	138.0	27.34	no
271	48.2	-61.40	-34.74	148.0	36.99	no
272	48.2	-61.40	-34.74	160.0	25.78	no
274	48.2	-61.40	-34.74	139.0	34.27	**yes**
275	48.2	-61.40	-34.74	146.0	27.63	no
276	48.2	-61.40	-34.74	156.0	29.30	no
277	48.2	-61.41	-34.76	131.0	34.41	no
278	48.2	-61.41	-34.76	143.0	31.56	no
279	48.2	-61.41	-34.76	135.0	36.21	no
280	48.2	-61.41	-34.76	171.0	27.97	no
281	48.2	-61.41	-34.76	146.0	31.38	no
282	48.2	-61.41	-34.76	170.0	30.41	**yes**
283	48.2	-61.41	-34.76	158.0	38.33	no
284	48.2	-61.41	-34.76	169.0	34.03	no
285	48.2	-61.41	-34.76	164.0	32.13	no
286	48.2	-61.41	-34.76	144.0	32.77	no
290	48.4	-59.95	-27.66	158.0	35.51	no
291	48.4	-59.95	-27.66	167.0	35.28	no
293	48.4	-59.95	-27.66	151.0	33.78	no
295	48.4	-59.87	-27.65	150.0	28.82	no
296	48.4	-59.87	-27.65	157.0	31.18	no
297	48.4	-59.87	-27.65	144.0	30.49	no
298	48.4	-59.87	-27.65	164.0	23.03	no
299	48.4	-59.87	-27.65	158.0	33.96	no
300	48.4	-59.87	-27.65	166.0	42.37	no
301	48.4	-59.87	-27.65	162.0	37.74	no
304	48.4	-59.05	-26.26	138.0	38.99	no
305	48.4	-59.05	-26.26	176.0	31.90	no

Table S2 (continued)

Sample	Management subarea	Latitude	Longitude	Length (cm)	Coverage	PCA outlier?
309	48.4	-59.05	-26.26	153.0	31.82	**yes**
322	48.4	-58.59	-26.07	161.0	27.24	no
332	48.4	-58.43	-26.02	178.0	31.76	no
335	48.4	-58.43	-26.02	149.0	30.16	no
337	48.4	-58.43	-26.02	185.0	29.10	no
339	48.4	-58.43	-26.02	141.0	31.21	no
342	48.4	-57.94	-26.29	141.0	32.88	no
348	48.4	-57.69	-26.29	123.0	33.46	no
349	48.4	-57.69	-26.29	150.0	32.43	no
353	48.4	-58.77	-25.25	152.0	31.34	no
354	48.4	-58.77	-25.25	149.0	29.27	no
357	48.4	-58.77	-25.25	167.0	36.29	no
358	48.4	-58.77	-25.25	160.0	34.54	no
388	48.4	-58.61	-25.04	148.0	33.50	no
391	48.4	-58.87	-25.21	150.0	31.78	no
399	48.4	-58.45	-25.98	144.0	39.70	no

Table S3. WGR sample metadata including type, management area and subarea, latitude, longitude, year of capture, estimated year of birth, cohort, total fish length (cm), final coverage, and dataset inclusion. Note that all tissue samples in the pre-2000 cohort were previously analyzed in Kuhn and Gaffney (2008). 2×, downsampled to 2×; 5×, downsampled to 5×; noDS, not downsampled; na, not available.

Sample	Type	Area	Subarea	Latitude	Longitude	Year of capture	Estimated birth year	Cohort	Length (cm)	Coverage	Dataset inclusion
9432	tissue	48	48.1	-62.00	-58.00	2001	1985	pre-2000	na	11.40	noDS, 2×, 5×
1	otolith	48	48.1	-63.24	-59.57	2006	2000	pre-2000	54.0	3.40	noDS, 2×
312	otolith	48	48.5	-74.64	-27.84	2013	2000	post-2000	123.0	12.46	noDS, 2×, 5×
313	otolith	48	48.5	-74.64	-27.84	2013	2000	post-2000	118.0	5.92	noDS, 2×, 5×
314	otolith	48	48.5	-74.64	-27.84	2013	2000	post-2000	103.0	13.27	noDS, 2×, 5×
315	otolith	48	48.5	-74.64	-27.84	2013	2000	post-2000	128.0	10.86	noDS, 2×, 5×
527	otolith	48	48.2	-61.56	-38.72	2018	2005	post-2000	151.0	6.76	noDS, 2×, 5×
67	otolith	48	48.1	-62.08	-53.55	2019	2005	post-2000	141.0	4.23	noDS, 2×
95	otolith	48	48.1	-62.16	-53.57	2019	2005	post-2000	140.0	1.13	noDS
164	otolith	48	48.2	-61.40	-34.74	2019	2005	post-2000	156.0	2.94	noDS, 2×
168	otolith	48	48.2	-61.40	-34.74	2019	2005	post-2000	158.0	6.92	noDS, 2×, 5×
231	otolith	48	48.4	-58.58	-26.12	2019	2005	post-2000	116.0	1.81	noDS
251	otolith	48	48.4	-58.77	-25.25	2019	2005	post-2000	167.0	3.04	noDS, 2×
9479	tissue	58	58.4.2	-67.20	44.00	2001	1985	pre-2000	na	6.43	noDS, 2×, 5×
398	otolith	58	58.4.2	-66.56	71.95	2019	2010	post-2000	86.0	3.59	noDS, 2×
402	otolith	58	58.4.2	-66.56	71.95	2019	2010	post-2000	70.0	4.84	noDS, 2×
408	otolith	58	58.4.2	-66.58	71.68	2019	2005	post-2000	136.5	2.46	noDS, 2×
412	otolith	58	58.4.2	-66.49	71.74	2019	2010	post-2000	105.0	6.88	noDS, 2×, 5×
419	otolith	58	58.4.2	-66.60	71.48	2019	2010	post-2000	89.5	12.03	noDS, 2×, 5×
433	otolith	58	58.4.2	-66.44	71.60	2019	2005	post-2000	122.5	11.07	noDS, 2×, 5×
435	otolith	58	58.4.2	-66.44	71.60	2019	2010	post-2000	98.0	3.28	noDS, 2×
9506	tissue	88	88.1	-77.00	179.00	2001	1985	pre-2000	na	7.29	noDS, 2×, 5×
9512	tissue	88	88.1	-77.00	179.00	2001	1985	pre-2000	na	22.13	noDS, 2×, 5×
9522	tissue	88	88.1	-73.00	171.00	2001	1985	pre-2000	na	9.52	noDS, 2×, 5×

Table S3 (continued)

Sample	Type	Area	Subarea	Latitude	Longitude	Year of capture	Estimated birth year	Cohort	Length (cm)	Coverage	Dataset inclusion
9525	tissue	88	88.1	-71.00	176.00	2001	1985	pre-2000	na	7.94	noDS, 2×, 5×
9540	tissue	88	88.1	-70.00	179.00	2001	1985	pre-2000	na	13.40	noDS, 2×, 5×
9544	tissue	88	88.1	-70.00	179.00	2001	1985	pre-2000	na	5.90	noDS, 2×, 5×
12821	tissue	88	88.2	-68.00	-122.00	2005	1990	pre-2000	na	3.43	noDS, 2×
12822	tissue	88	88.2	-68.00	-122.00	2005	1990	pre-2000	na	1.76	noDS
12836	tissue	88	88.2	-68.00	-122.00	2005	1990	pre-2000	na	2.32	noDS, 2×
12840	tissue	88	88.2	-68.00	-123.00	2005	1990	pre-2000	na	2.29	noDS, 2×
12841	tissue	88	88.2	-68.00	-123.00	2005	1990	pre-2000	na	2.77	noDS, 2×
1-425	tissue	88	88.1	-64.00	178.00	2019	2005	post-2000	148.0	15.37	noDS, 2×, 5×
33-429	tissue	88	88.1	-72.00	181.00	2019	2005	post-2000	156.0	13.09	noDS, 2×, 5×
34-430	tissue	88	88.1	-72.00	181.00	2019	2005	post-2000	142.0	11.82	noDS, 2×, 5×
35-431	tissue	88	88.1	-72.00	181.00	2019	2005	post-2000	152.0	8.78	noDS, 2×, 5×
4-456	tissue	88	88.1	-65.00	176.00	2019	2005	post-2000	145.0	19.92	noDS, 2×, 5×
6-450	tissue	88	88.1	-65.00	178.00	2019	2005	post-2000	142.0	2.88	noDS, 2×
38-434	tissue	88	88.1	-73.00	180.00	2020	2005	post-2000	134.0	1.72	noDS
42-438	tissue	88	88.1	-73.00	180.00	2020	2005	post-2000	127.0	11.28	noDS, 2×, 5×
50-446	tissue	88	88.1	-73.00	180.00	2020	2005	post-2000	158.0	27.09	noDS, 2×, 5×

Table S4. Weighted mean F_{ST} values between various groupings: CCAMLR management areas (48, 58 and 88), coverage levels (medium 5×–10×, high >10×), cohort (pre-2000, post-2000), and sex (male, female and unknown) based on genotype likelihoods from the 5× dataset.

Comparison	Weighted mean F_{ST}
CCAMLR management area 48 vs 58	0.0487
CCAMLR management area 48 vs 88	0.0390
CCAMLR management area 58 vs 88	0.0430
medium (5×–10×) vs high (>10× coverage)	0.0218
pre-2000 vs post-2000 cohort	0.0304
male vs female	0.0245
male vs unknown sex	0.0323
female vs unknown sex	0.0337

Supplementary Figures

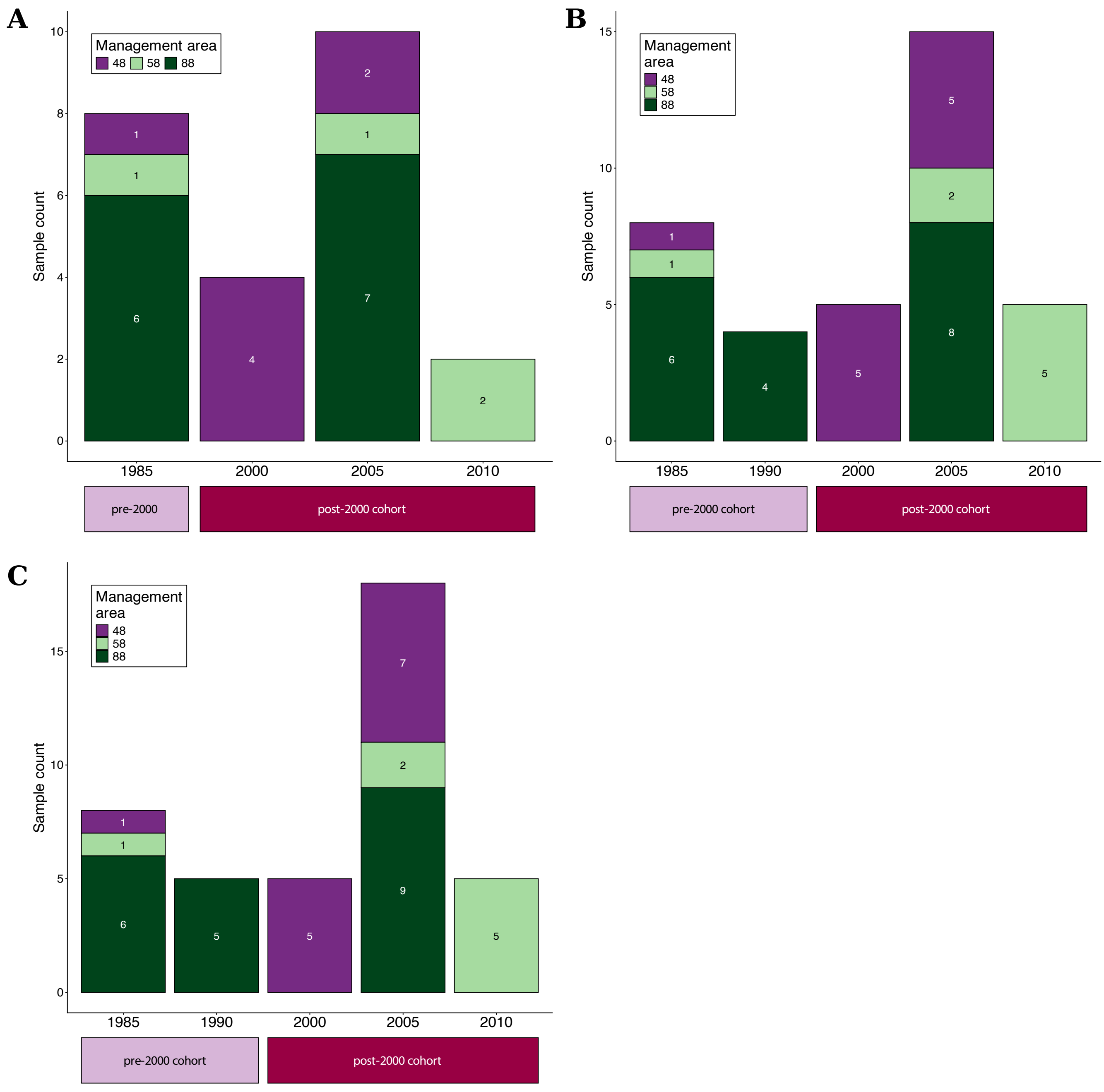

Figure S1. Sample counts across cohorts and management areas from (A) 5× (n = 24), (B) 2× (n = 37) and (C) noDS (n = 41) datasets.

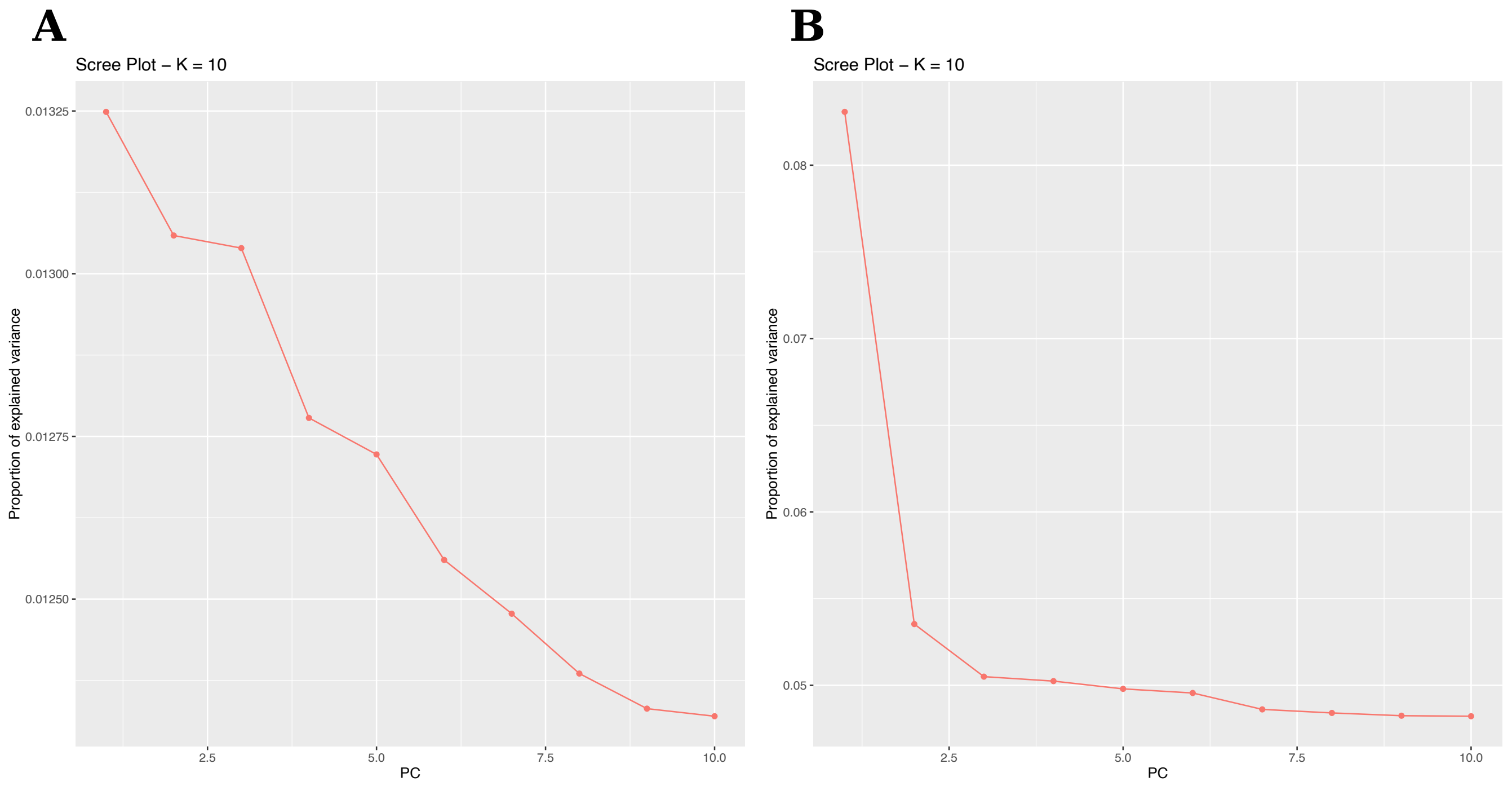

Figure S2. Scree plots generated in PCADAPT supporting the use of 2 principal components (K) for outlier scans based on (A) 3RADseq and (B) WGR data (adapted from Caccavo et al. (2024), Figure S1).

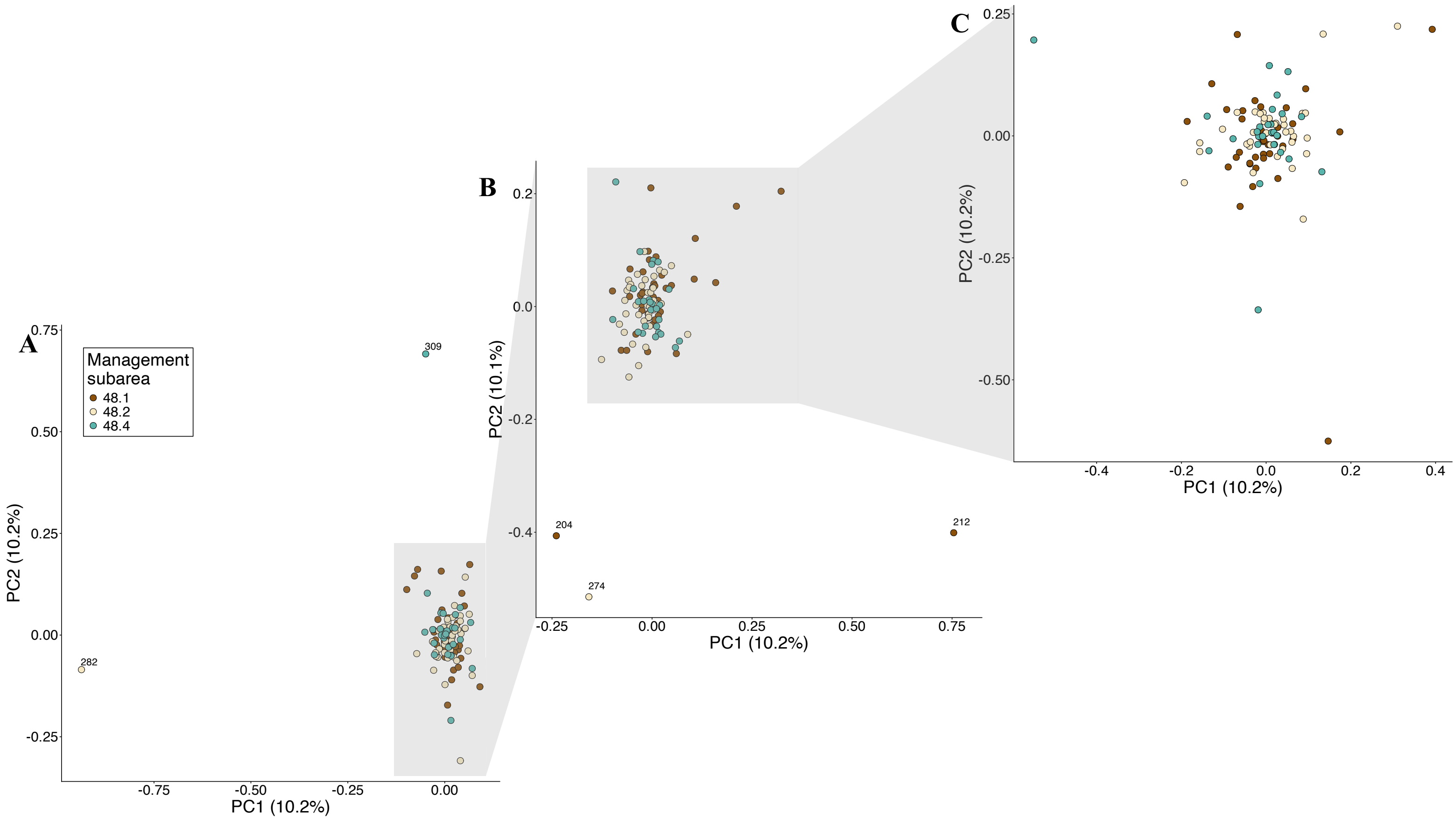

Figure S3. PCA based on 3RADseq data of *D. mawsoni* in the Weddell Sea. Groups labeled by CCAMLR management subarea (48.1, 48.2, 48.3). (A) Original PCA *n* = 109 samples seen in Figure 3a highlighting samples under the gray box used in the subsequent PCA of (B) *n* = 106 samples, with 2 outliers (samples 282 and 309) removed, highlighting samples under the gray box used in the final PCA of (C) *n* = 103 samples, with an additional 3 outliers removed (samples 282 and 309 as well as samples 204, 274, and 212). Data labels indicate sample IDs.

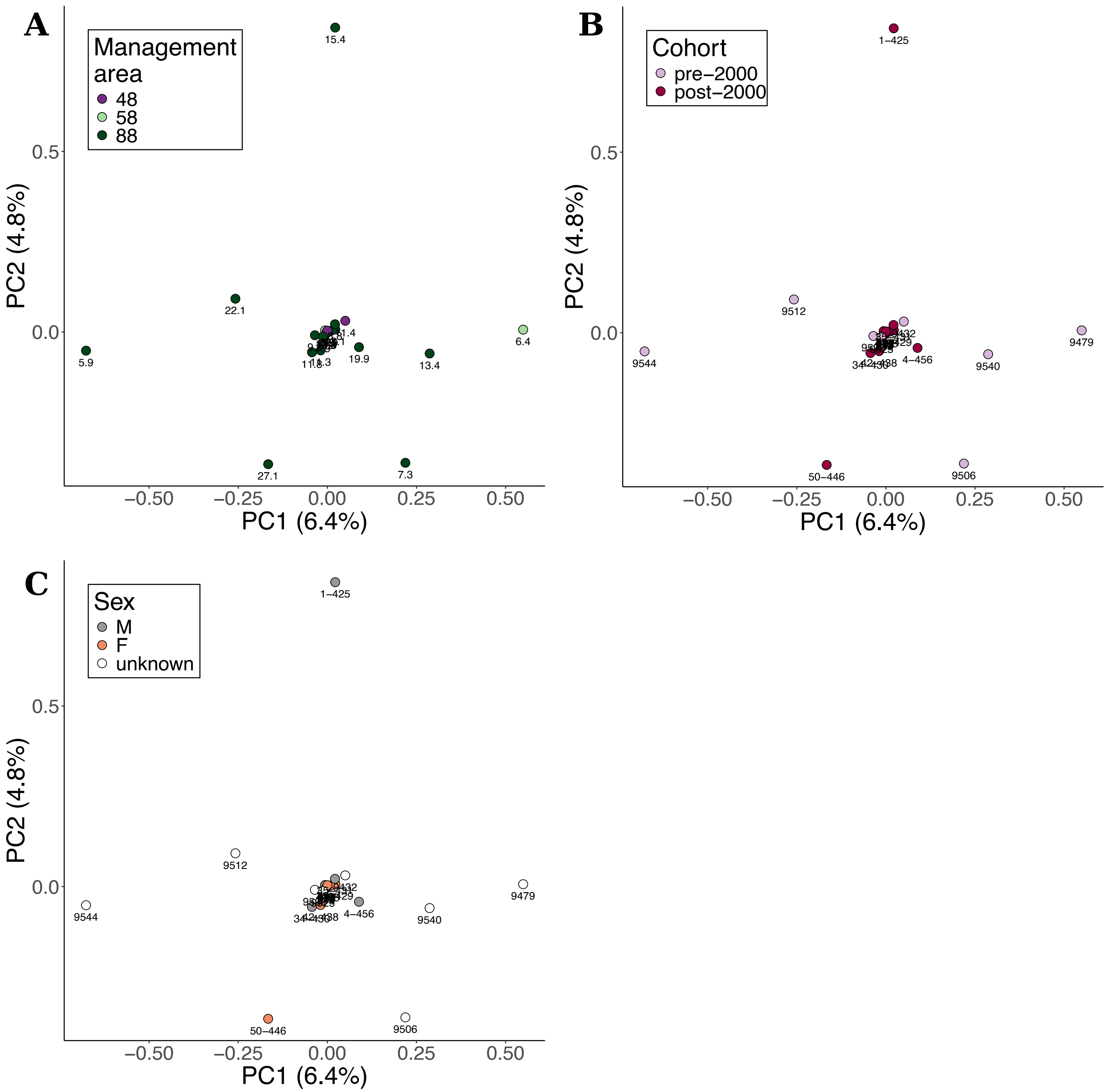

Figure S4. PCA plots of alternative sample groupings based on WGR data (5× dataset, n = 24). (A) CCAMLR management area (48, 58, 88), data labels indicate coverage (×). (B) Cohort, data labels indicate sample ID. (C) Sex, data labels indicate sample ID.

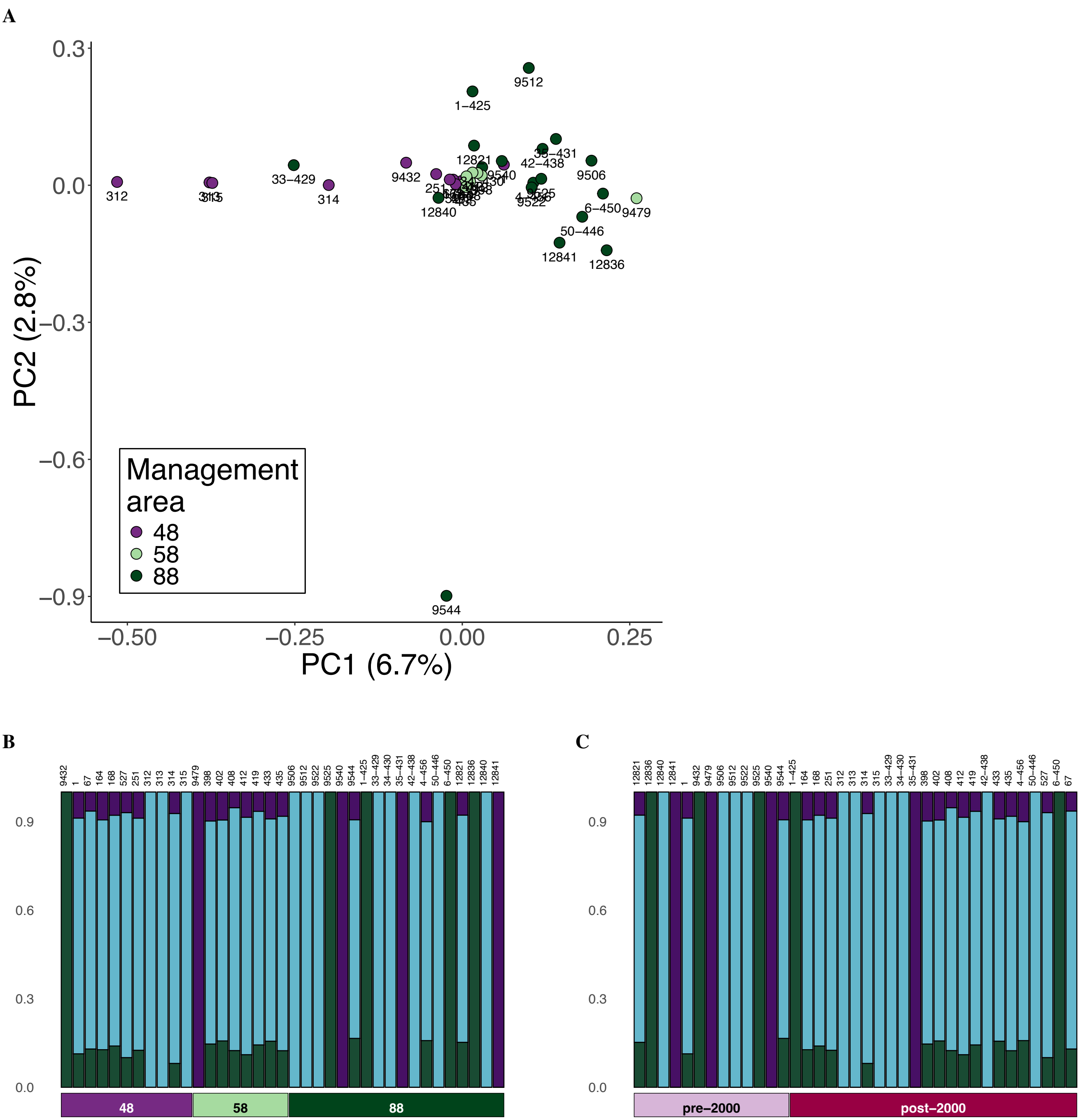

Figure S5. Circumpolar population structure of *D. mawsoni* based on WGR data (2× dataset, *n* = 37). Groups labeled by CCAMLR management areas (48, 58, 88). (A) PCA based on all samples, data labels indicate sample IDs. Admixture plot of population structure for $K = 3$, with sample IDs listed above the plot; ordered by (B) CCAMLR area. (C) cohort.

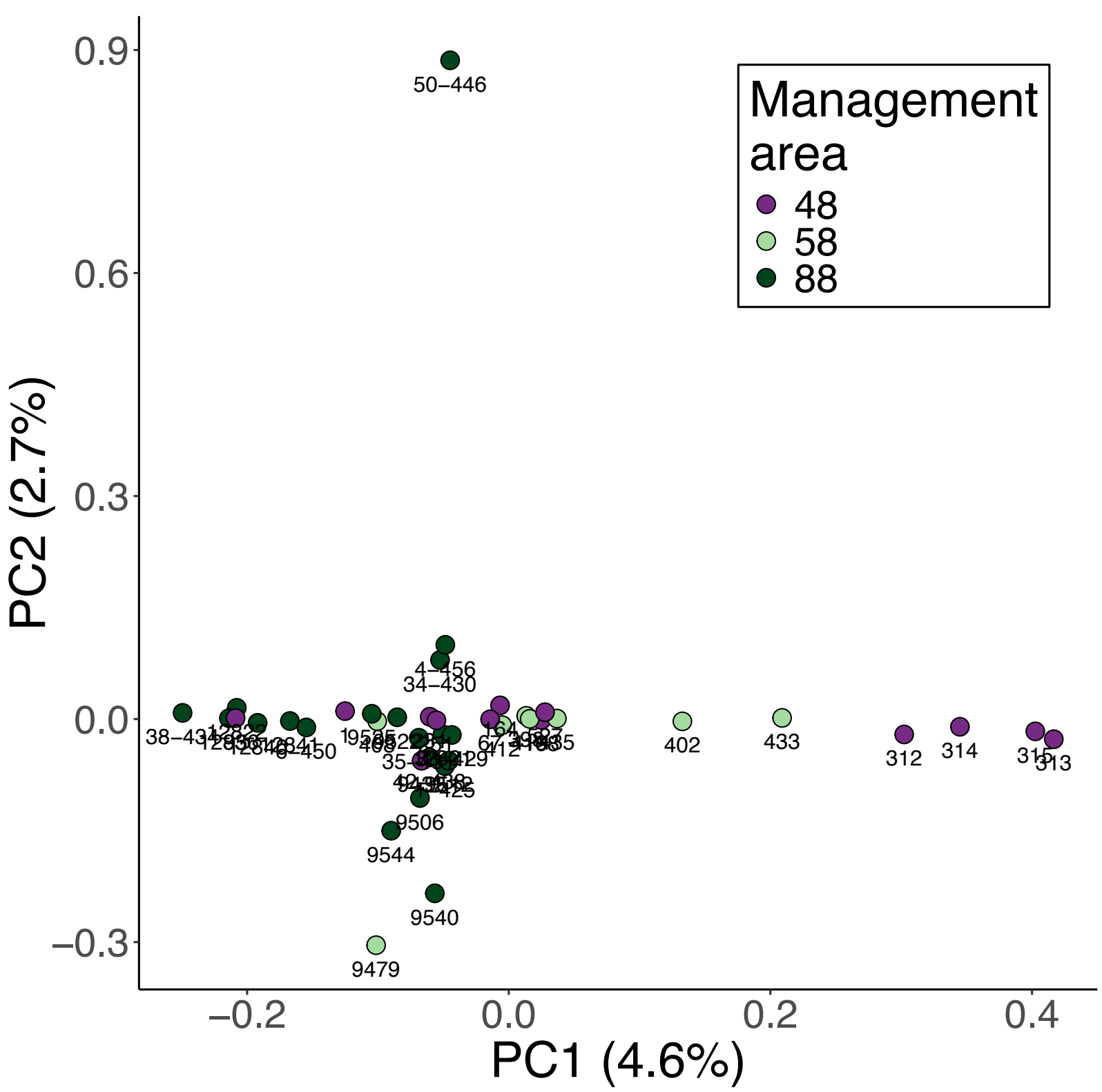

Figure S6. Circumpolar population structure of *D. mawsoni* based on WGR data (noDS dataset, $n = 41$). Groups labeled by CCAMLR management areas (48, 58, 88). PCA based on all samples, data labels indicate sample IDs.

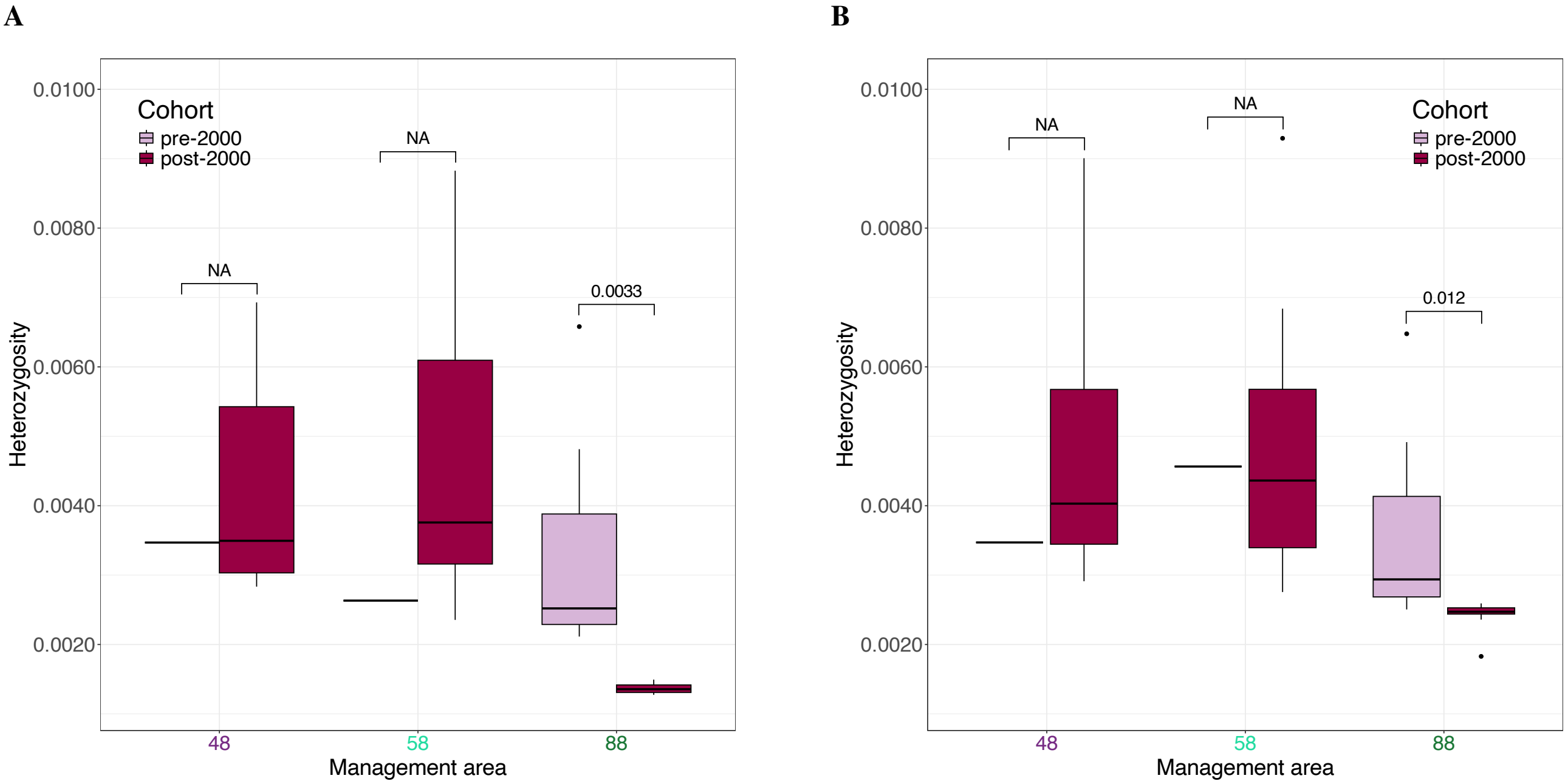

Figure S7. *D. mawsoni* heterozygosity from all three circumpolar management areas including across cohorts based on (A) 2× (n = 37) and (B) noDS (n = 41) datasets. P-values from post hoc pairwise t-tests are shown. Where NA indicated, post hoc tests were not possible due to groups with n = 1 individual.

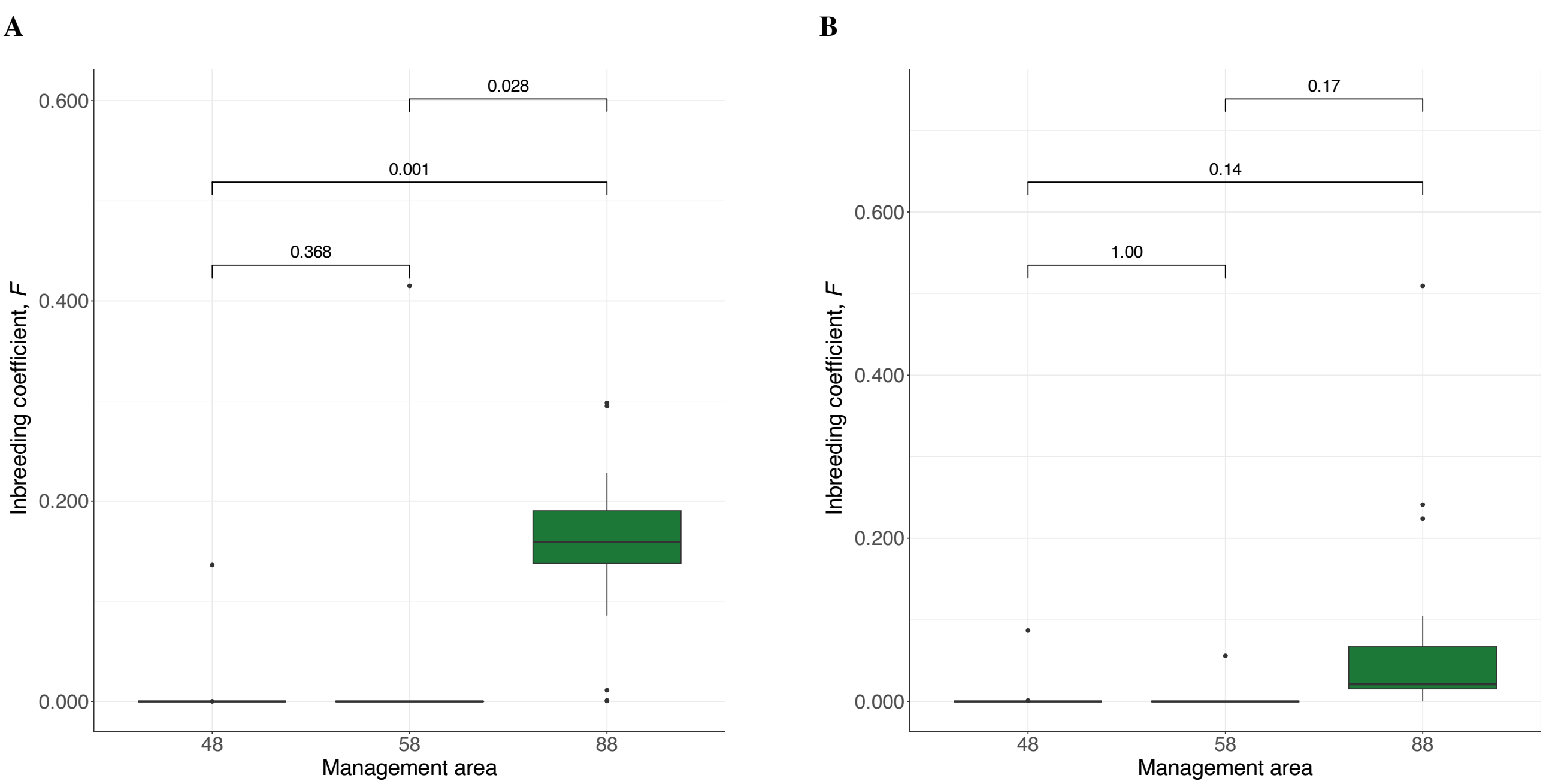

Figure S8. *D. mawsoni* individual inbreeding coefficients, F, based on (A) 2× (n = 37) and (B) noDS (n = 41) datasets. P-values from post hoc pairwise t-tests are shown.

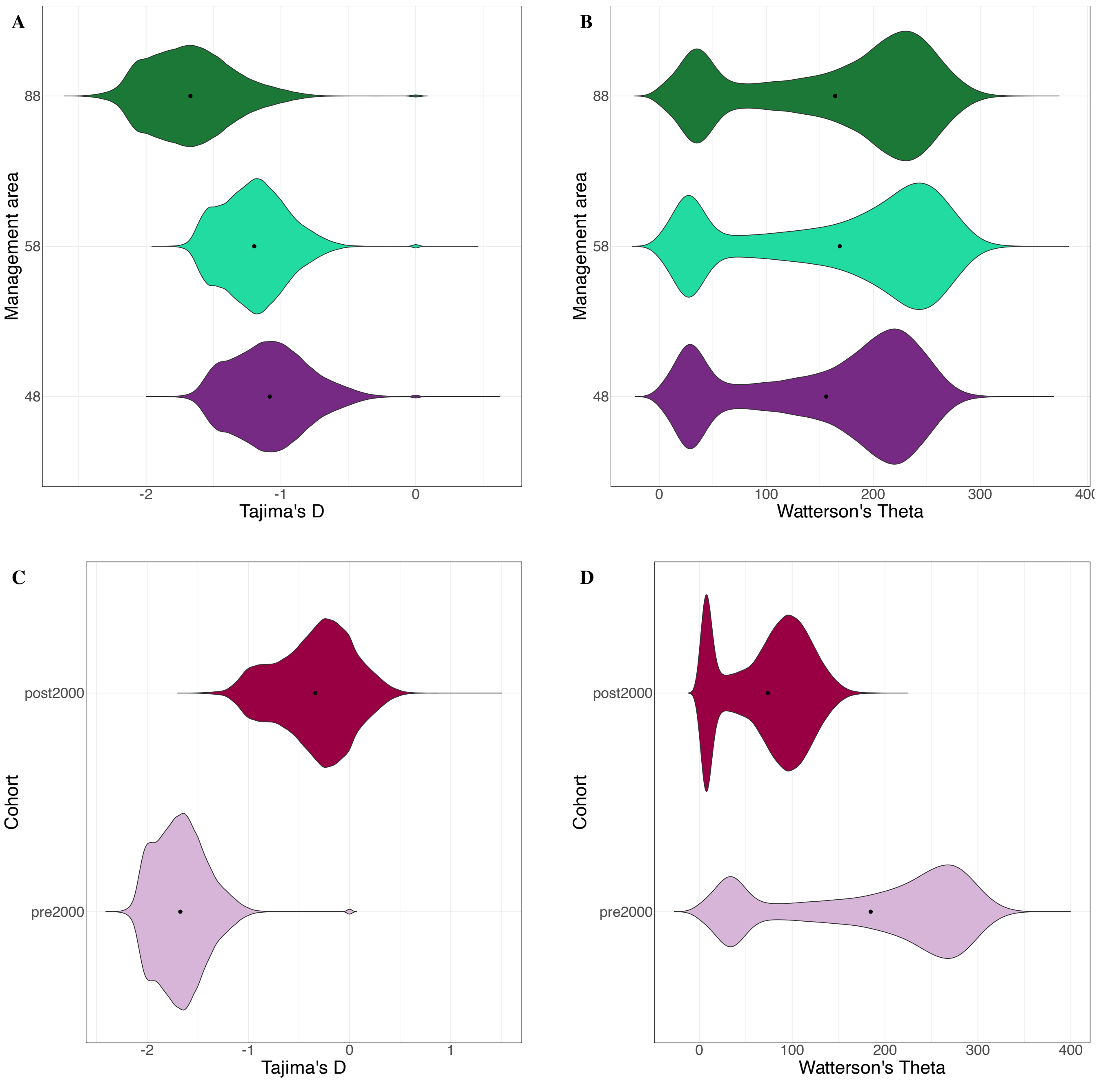

Figure S9. Violin plots of Tajima's D and Watterson's theta estimates derived from *D. mawsoni* genotype-likelihoods (2× dataset) using a sliding window approach with a window size of 50 kb and a step size of 10 kb; black dots indicate the mean value across the 50 kb-windows. (A) Tajima's D and (B) Watterson's theta estimates across CCAMLR management areas (n = 37). (C) Tajima's D and (D) Watterson's theta estimates between cohorts within area 88 (n = 18).

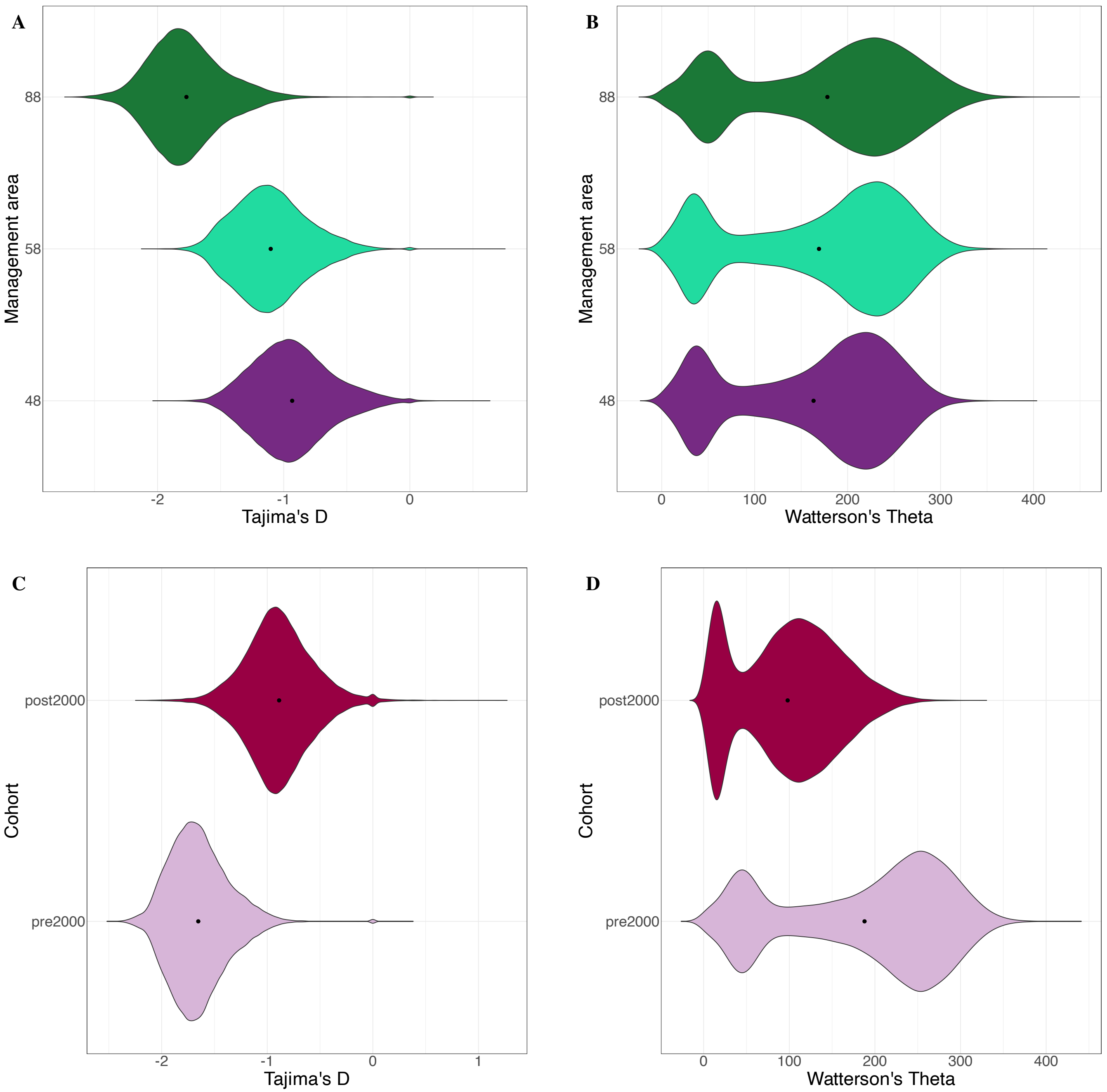

Figure S10. Violin plots of Tajima's D and Watterson's theta estimates derived from *D. mawsoni* genotype-likelihoods (noDS dataset) using a sliding window approach with a window size of 50 kb and a step size of 10 kb; black dots indicate the mean value across the 50 kb-windows. (A) Tajima's D and (B) Watterson's theta estimates across CCAMLR management areas (n = 41). (C) Tajima's D and (D) Watterson's theta estimates between cohorts within area 88 (n = 20).
